# Excluding Ascites From the GEMA‐Na Score Does Not Impact Outcome Predictions in Liver Transplant Candidates

**DOI:** 10.1111/liv.70520

**Published:** 2026-01-29

**Authors:** Manuel Luis Rodríguez‐Perálvarez, Antonio Manuel Gómez‐Orellana, Avik Majumdar, Geoffrey W. McCaughan, María Kalafateli, Rhiannon Taylor, Gloria de la Rosa, María Victoria Aguilera, Mikel Gastaca, Carmen Cepeda‐Franco, María Luisa Ortiz, Jordi Colmenero, Alejandra Otero, Rocío González Grande, Alba Cachero, Esther Molina Pérez, Mónica Barreales, Rosa Martín Mateos, María Rodríguez‐Soler, Mario Romero, Cristina Dopazo, Carmen Alonso Martín, Elena Otón, Luisa González Diéguez, María Dolores Espinosa, Ana Arias Milla, Gerardo Blanco Fernández, Sara Lorente, Antonio Cuadrado Lavín, Miguel Sogbe, David Guijo‐Rubio, César Hervás Martínez, Emmanuel Tsochatzis

**Affiliations:** ^1^ Department of Hepatology and Liver Transplantation Hospital Universitario Reina Sofía, IMIBIC Córdoba Spain; ^2^ Centro de Investigación Biomédica en Red de Enfermedades Hepáticas y Digestivas (CIBERehd) Madrid Spain; ^3^ Department of Computer Science and Artificial Intelligence Universidad de Córdoba Córdoba Spain; ^4^ Victorian Liver Transplant Unit and Australian Centre for Transplantation Excellence and Research Austin Health Melbourne Australia; ^5^ University of Melbourne Melbourne Australia; ^6^ AW Morrow Gastroenterology and Liver Centre and Australian National Liver Transplant Unit Royal Prince Alfred Hospital Sydney Australia; ^7^ Central Clinical School The University of Sydney Sydney Australia; ^8^ Department of Gastroenterology General Hospital of Patras Patras Greece; ^9^ Department of Statistics and Clinical Studies NHS Blood and Transplant Bristol UK; ^10^ Organización Nacional de Trasplantes (ONT) Madrid Spain; ^11^ Department of Hepatology and Liver Transplantation Hospital La Fe e Instituto de Investigación Sanitaria La Fe Valencia Spain; ^12^ Department of HPB Surgery and Liver Transplantation Hospital Universitario de Cruces Bilbao Spain; ^13^ Department of HPB Surgery and Liver Transplantation Hospital Virgen del Rocío Sevilla Spain; ^14^ Department of Hepatology and Liver Transplantation Hospital Universitario Virgen Arrixaca Murcia Spain; ^15^ Liver Unit, Hospital Cínic, IDIBAPS University of Barcelona Barcelona Spain; ^16^ Department of Hepatology and Liver Transplantation Centro Hospitalario Universitario de A Coruña A Coruña Spain; ^17^ Department of Hepatology and Liver Transplantation Hospital Regional Universitario de Málaga Málaga Spain; ^18^ Department of Liver Transplantation Hospital Universitario de Bellvitge L’Hospitalet de Llobregat Spain; ^19^ Department of Liver Transplantation Centro Hospitalario Universitario de Santiago Santiago de Compostela Spain; ^20^ Department of Hepatology and Liver Transplantation Hospital Universitario 12 de Octubre Madrid Spain; ^21^ Department of Liver Transplantation, Hospital Universitario Ramón y Cajal IRYCIS, Universidad de Alcalá Madrid Spain; ^22^ Department of Hepatology and Liver Transplantation Hospital General Universitario Dr. Balmis de Alicante, ISABIAL Alicante Spain; ^23^ Department of Hepatology and Liver Transplantation Hospital General Universitario e Instituto de Investigación biomédica Gregorio Marañón Madrid Spain; ^24^ Department of HPB Surgery and Transplantation, Vall d'hebron Hospital Universitari, VHIR Universitat Autónoma de Barcelona Barcelona Spain; ^25^ Department of Hepatology and Liver Transplantation Hospital Rio Hortega Valladolid Spain; ^26^ Department of Hepatology and Liver Transplantation Hospital Virgen de la Candelaria Santa Cruz de Tenerife Spain; ^27^ Department of Hepatology and Liver Transplantation Hospital Universitario Central de Asturias Oviedo Spain; ^28^ Department of Hepatology and Liver Transplantation Hospital Virgen de Las Nieves Granada Spain; ^29^ Department of Hepatology and Liver Transplantation Hospital Universitario Puerta de Hierro Madrid Spain; ^30^ Department of Liver Transplantation Hospital Universitario de Badajoz Badajoz Spain; ^31^ Department of Hepatology and Liver Transplantation, Hospital Universitario Lozano Blesa Instituto de Investigaciones Sanitarias de Aragón (IIS Aragón) Zaragoza Spain; ^32^ Department of Hepatology and Liver Transplantation Hospital Universitario Marqués de Valdecilla, IDIVAL Santander Spain; ^33^ Liver Unit and HPB Oncology Area Clinica Universidad de Navarra Pamplona Spain; ^34^ Sheila Sherlock Liver Unit and UCL Institute for Liver and Digestive Health Royal Free Hospital London UK

**Keywords:** allocation, cirrhosis, equity, gender, MELD 3.0, mortality

## Abstract

**Background and Aims:**

Although GEMA‐Na outperforms MELD 3.0 for liver allocation, concerns about the subjectivity of its ascites component persist. We compared the performance of a GEMA‐Na iteration that excludes ascites with other allocation scores.

**Approach and Results:**

A multinational cohort study was conducted, including adult candidates for elective liver transplantation in the UK (2010–2020), Australia (1998–2020), and Spain (2016–2021). The primary outcome was mortality or delisting for sickness within 90 days. The prognostic impact of ascites was evaluated using multivariable Cox's regression. Discrimination was assessed using Harrell's c‐statistics (Hc). The study included 15 391 patients (28.5% women). The prevalence of the primary outcome was 5.8% in the UK, 5.3% in Australia, and 4.7% in Spain. The presence and severity of ascites was associated with an incremental risk of the primary outcome: 3.3% without ascites, 5.8% with mild ascites, and 7.7% with moderate–severe ascites (*p* < 0.001). Removal of ascites from the GEMA‐Na score resulted in a one‐point reduction in 18% of patients (52.4% of patients with moderate–severe ascites). GEMA‐Na without ascites showed only a marginal decrease in discrimination (Hc = 0.755 vs. Hc = 0.753; *p* = 0.007) but still significantly outperformed MELD 3.0 (Hc = 0.734; *p* < 0.001) and MELD‐Na (Hc = 0.737; *p* < 0.001). In women, GEMA‐Na with and without ascites demonstrated comparable discrimination (Hc = 0.784 vs. Hc = 0.783; *p* = 0.61), both outperforming MELD 3.0 (Hc = 0.750; *p* < 0.001), and MELD‐Na (Hc = 0.749; *p* < 0.001).

**Conclusions:**

Despite the prognostic impact of ascites among liver transplant candidates, GEMA‐Na without ascites outperformed other scores in predicting wait‐list outcomes and may be used wherever the inclusion of ascites is considered too subjective.

## Abstract (highlights)

**Key Points**

- Among patients with end‐stage liver disease listed for liver transplantation, the presence and grade of ascites are associated with an incremental risk of 90‐day mortality or delisting for sickness.
- Including ascites as part of a transplant prioritisation score such as GEMA‐Na raises concerns on subjectivity in some scenarios.
- An iteration of GEMA‐Na without ascites results in minimal score changes and roughly 80% of patients retain the same score.
- In more than 15.000 patients from three different countries, GEMA‐Na without ascites consistently outperformed MELD‐Na and MELD 3.0 in predicting wait‐list outcomes, including subgroups of interest such as women and patients with moderate–severe ascites.

**Abbreviations**
95% CI: 95% confidence interval
GEMA‐Na: gender‐equity model for liver allocation corrected by serum sodium
Hc: Harrell's c‐statistic
HR: hazard ratio
IQR: interquartile range
LT: liver transplantation
MELD‐Na: model for end‐stage liver disease corrected by serum sodium
MELD 3.0: model for end‐stage liver disease 3.0
NHS: National Health Service (United Kingdom)
ONT: *Organización Nacional de Trasplantes* (Spain)
RFH‐GFR: royal free hospital glomerular filtration rate
UK: United Kingdom

## 1 Introduction

The imbalance between the number of donors and potential candidates for liver transplantation (LT) requires the implementation of prioritisation policies which grant earlier access to transplant for the sickest patients. The model for end‐stage liver disease (MELD) was adopted in the United States two decades ago and allowed a dramatic decrease of waiting‐list mortality [1]. In 2008, MELD had its most relevant update by incorporating serum sodium to the formula (MELD‐Na) [2]. Most LT allocation systems nowadays are based on MELD or MELD‐Na [3], but a progressive decline of their discrimination capacity has been documented [4]. In addition, since the implementation of MELD, women are disadvantaged to access LT and show higher risk of mortality or delisting due to clinical deterioration [5, 6]. Although sex disparities for accessing LT are likely multifactorial, the main culprit is serum creatinine as part of the MELD and MELD‐Na scores: with identical renal function, women receive less creatinine‐derived score points due to reduced body size and muscle mass on average as compared with men [7, 8].

Two newly created scores aimed to correct sex disparities for accessing LT are recommended by recent international guidelines for waiting‐list prioritisation [9]. MELD 3.0, which was developed and validated in the United States, incorporated sex and serum albumin into the MELD‐Na formula and controlled relevant interactions. Serum creatinine was kept, although capped at 3 mg/dL [10]. On the other hand, the gender‐equity model for liver allocation corrected by serum sodium (GEMA‐Na) was trained and internally validated in the United Kingdom (UK), and externally validated in Australia [11], Spain [6], and Italy [12]. GEMA‐Na replaced serum creatinine with the Royal Free Hospital glomerular filtration rate (RFH‐GFR) [13], with re‐weighting and re‐fitting of the remaining MELD‐Na components [11, 14]. Although GEMA‐Na allowed more accurate predictions of waiting‐list outcomes than MELD 3.0 in several countries with different allocation systems, particularly among women, it has been criticised for incorporating moderate–severe ascites to calculate RFH‐GFR. Although ascites is an objective parameter in patients requiring regular large‐volume paracentesis or based on imaging, there is an element of subjectivity if the assessment of moderate ascites is based on clinical examination. This is deemed problematic as misdiagnosed ascites could artificially increase the transplant priority score, thus introducing bias in organ allocation.

In the present multinational cohort study, we aimed to compare the performance of an iteration of GEMA‐Na without ascites with the original GEMA‐Na model, MELD 3.0, and MELD‐Na in predicting waitlist outcomes in candidates for LT.

## 2 Materials and Methods

### 2.1 Study Population, Data Sources and Ethical Considerations

This is an observational multicentre study including three cohorts of adult patients consecutively enlisted for elective LT in the UK, Spain, and Australia, respectively. In the UK, data from transplant candidates listed in any of the seven LT institutions across the country from April 2010 to March 2020 were obtained from the UK Transplant Registry, held by the National Health Service (NHS) Blood and Transplant. In Spain, data of patients listed from January 2016 to December 2021 were obtained from the *Organización Nacional de Trasplantes* (ONT), which depends on the Spanish Ministry of Health and collects information from 25 adult LT institutions. The third study cohort comprised data from the two largest Australian LT institutions, namely Royal Prince Alfred Hospital (Australian National Liver Transplant Unit) and Austin Hospital (Victorian Liver Transplant Unit), from January 1998 to December 2020. In all cohorts, exclusion criteria were acute liver failure listed for urgent LT, living donation, combined organ transplantation, retransplantation, non‐cirrhotic special indications for LT, or missing values precluding the calculation of GEMA‐Na. The limited number of variables required for model calculations and outcomes allowed < 5% of missing data in the present study so we used a ‘complete‐case analysis’ approach. We reported missing data, if any, in each analysis.

The study complies with the Declaration of Helsinki. Before sharing their corresponding anonymized databases, the study protocol was evaluated and approved by the NHS Blood and Transplant authority (UK), the ONT (Spain), and the Sydney Local Health District Human Research Ethics Committee (Australia). In addition, the study protocol was approved by the Andalusian ethics committee, which serves as the reference committee for the institution where the data analysis was conducted (reference 5408, 2022).

### 2.2 Definitions, Variables and Predicting Scores

Age, sex assigned at birth, aetiology of liver disease, reason for being included in the waiting‐list, date of inclusion, events on the waiting‐list (death, removal from the waiting‐list and transplantation) and date of such events were retrieved from the original datasets.

Predicting scores were calculated at inclusion in the waiting‐list and all were ranked 6–40. Serum bilirubin, international normalised ratio (INR), serum sodium and serum creatinine were combined to calculate MELD‐Na according to established formulae [2]. Serum albumin and sex were added to the above to calculate MELD 3.0 (online calculator available at https://medcalculators.stanford.edu/meld) [10]. To calculate GEMA‐Na, we combined serum bilirubin, INR, serum sodium and the RFH‐GFR [11], which in turn comprised age, sex, serum urea, serum creatinine, INR, serum sodium and moderate–severe ascites as a binary variable [13] (online calculator available at https://gema‐transplant.com/home). Moderate–severe ascites at waiting‐list inclusion was considered if the patient required a large‐volume paracentesis within the previous four weeks or if it was clinically evident in the physical examination and confirmed with an imaging technique [15].

Finally, for calculating the iteration of GEMA‐Na without ascites, all patients were assigned a value of ‘ascites = 0’ so that this variable would not influence the RFH‐GFR calculation. The coefficients of the remaining variables of GEMA‐Na were unchanged. The formulas of RFH‐GFR, GEMA and GEMA‐Na without ascites such configured are shown below:

$$\begin{array}{c} \mathrm{RFH}-\mathrm{GFR}\;\text{without ascites}=45.9^{*}\;{\left({\text{creatinine}}^{-0.836}\right)}^{*}\\ \;{\left({\text{urea}}^{-0.229}\right)}^{*}\;{\left({\mathrm{INR}}^{-0.113}\right)}^{*}\;{\left({\mathrm{age}}^{-0.129}\right)}^{*}\;{\left({\text{sodium}}^{0.972}\right)}^{*}\;1.236\;\left(\text{if male}\right) \end{array}$$

$$\begin{array}{c} \text{GEMA without ascites}=3.777^{*}\ln\;\left(\text{Bilirubin}\right)+7.883^{*}\\ \ln\;\left(\mathrm{INR}\right)–8.306^{*}\ln\;\left(\mathrm{RFH}-\mathrm{GFR}\;\text{without ascites}\right)+31.932 \end{array}$$

$$\begin{array}{c} \text{GEMA}-\mathrm{Na}\;\text{without ascites}=\text{GEMA without ascites}-\mathrm{Na}\\ -\left[0.025^{*}\;{\text{GEMA without ascites}}^{*}\;\left(140-\mathrm{Na}\right)\right]+140 \end{array}$$

### 2.3 Outcomes and Sensitivity Analyses

The primary outcome of the study was mortality or exclusion from the waiting‐list due to clinical deterioration beyond transplant suitability within the first 90 days as a time‐dependent event, aligning with previous studies [2, 10, 11]. Outcome data were right‐censored at 90 days after the inclusion, or earlier than that time point if the patient underwent LT or was excluded for reasons other than clinical deterioration. Right‐censoring was used due to its suitability to analyse a score rank to predict mortality in the absence of transplant aligning with previous studies [10]. We did not implement a competing risk‐analysis considering transplantation as a competing event since it would be relevant to analyse the survival probability in the presence of transplant.

The analyses were performed in each cohort separately as these correspond to independent transplant environments with specific prioritisation policies. Sensitivity analyses were performed in pre‐defined subgroups of interest, namely women and patients with moderate–severe ascites.

### 2.4 Sample Size Calculation

We estimated the minimum sample size required to compare the discrimination between the Gender‐Equity Model for Liver Allocation corrected by serum sodium (GEMA‐Na) and its iteration without ascites to predict mortality or delisting for sickness within the first 90 days from listing. We followed the method proposed by Jinks et al. which was specifically designed for multivariate prognostic models for time‐to‐event data considering right‐censoring [16]. The authors considered for their formulae the Royston & Sauerbrei D measure of discrimination [17], which could be derived from the Harrell c‐index. The expected results on discrimination of GEMA‐Na and the prevalence of the primary outcome were obtained from the external validation cohort of the study by Rodríguez‐Perálvarez et al. [6]. Since no previous study evaluated the performance of GEMA‐Na without ascites, we assumed a similar performance as that observed with MELD 3.0 in the above referred study. The following assumptions were made:

– Expected D statistic of GEMA‐Na: 1.9244658, standard error (D) 0.2044043
– Expected D statistic of GEMA‐Na without ascites: 1.79807867, standard error (D) 0.20447317
– Expected proportion of patients experiencing the primary outcome: 5.5%
– Statistical power: 90%
– Proportion of censoring: 50%
– Alpha error: 0.05.

Under these premises the minimum sample size would be 4932 patients, including 271 patients experiencing the primary outcome. The present study comprised 15 391 patients, including 822 patients experiencing the primary outcome. Regarding subgroups of interest, there were 4384 women and 5121 patients with moderate–severe ascites.

### 2.5 Performance of the Models and Statistical Analysis

Firstly, we tested the performance of the RFH‐GFR model without ascites in assessing GFR using the patient cohort from the original publication [13], in order to establish that it still provides an accurate estimation of renal function (supplementary text, Table S1 and Figures S1 and S2). We subsequently used the RFH‐GFR equation without ascites in the GEMA score for all included patients. Predictive models were assessed in terms of discrimination, which refers to the ability of the model to differentiate between patients who experienced the primary outcome and those who did not. Discrimination was assessed using Harrell's c‐statistic (Hc) and its 95% confidence interval (95% CI), which is specific for time‐dependent outcomes with right‐censoring. The statistical comparison of discrimination among different models was performed using a one‐shot nonparametric approach which does not require resampling, as described by Kang et al. [18]. The Brier score, calculated as the mean squared error of predicted probabilities, was used to measure the overall accuracy of the scores. More accurate predictions would yield lower Brier scores.

The iteration of GEMA‐Na without ascites was tested for calibration, which informs about the homogeneity of the predictions across the disease severity spectrum. Calibration was assessed by the goodness‐of‐fit after stratifying the population in deciles of risk as proposed by Greenwood‐Nam‐D’Agostino [19]. If the number of predicted events in a certain decile was < 2, contiguous deciles were merged to allow the analysis. To visualise the impact of transitioning from GEMA‐Na with ascites to GEMA‐Na without ascites on the waiting‐list composition, we analysed clinical features and outcomes of reclassified patients, defined as those showing a change of ≥ 1 score point, either upgraded or downgraded in the list. Finally, we provided decision‐curve analyses for each model showing the net benefit against the threshold probability of the primary outcome.

Continuous variables were expressed as mean and standard deviation, except for those with an asymmetric distribution, in which median and interquartile range (IQR) were used. Categorical variables were displayed as absolute numbers and percentages. The appropriate contrast tests were used according to the type of variable involved in the analysis. Kaplan–Meier curves (log‐rank test) and multivariable Cox's regression were used to analyse the effect of ascites on mortality or exclusion from the waiting‐list due to clinical deterioration. Risk estimates were expressed as hazard ratio (HR) and their 95% CI. A bilateral *p* < 0.05 was considered statistically significant. Analyses were performed by using R v4.2.1 (RStudio‐Posit software, Boston, USA) and SPSS 27.0 (IBM, Chicago, USA).

## 3 Results

### 3.1 Study Cohorts and Outcomes in the Waiting‐List

The study population comprised 15 391 patients, of whom 4384 were women (28.5%) from three cohorts: UK (*n* = 7682), Spain (*n* = 6071) and Australia (*n* = 1638). Clinical features and waiting‐list outcomes in each cohort are presented in Table 1. Women accounted for 33.6% of patients in the UK, 26.4% in Australia, and 22.6% in Spain. The most frequent aetiology of liver disease was alcohol‐related in the UK and Spain (36.2% and 49.8%, respectively), and chronic hepatitis C in Australia (41.6%). The prevalence of moderate–severe ascites at inclusion on the waiting‐list was 31.4% in the UK, 35% in Spain, and 35.5% in the Australian cohort. Rates of transplantation were highest in Spain (85.5%), followed by Australia (79.8%) and the UK (76.7%). All prioritisation scores at inclusion were higher in the UK and Australian cohorts compared with the Spanish cohort. Mortality or delisting due to clinical deterioration within the first 90 days was 5.8% in the UK, 5.3% in Australia and 4.7% in Spain.

**TABLE 1:** Clinical features and outcomes of 15 391 adult patients enlisted for elective liver transplantation in the United Kingdom (2010–2020), Spain (2016–2021), and in two Australian institutions (1998–2020).

VARIABLE	UNITED KINGDOM (*n* = 7682)	SPAIN (*n* = 6071)	AUSTRALIA (*n* = 1638)
Age (years)	53.22 ± 11.55	57.81 ± 8.60	53.52 ± 9.28
Sex (women)	2578 (33.6%)	1374 (22.6%)	432 (26.4%)
Height (cm)	N/A	168.42 ± 8.58	N/A
Aetiology (alcohol)	2783 (36.2%)	3026 (49.8%)	474 (28.9%)
Aetiology (hepatitis C)	1242 (16.2%)	1325 (21.8%)	681 (41.6%)
Aetiology (MASH/cryptogenic)	1374 (17.9%)	350 (5.8%)	203 (12.4%)
Aetiology (PBC)	633 (8.2%)	175 (2.9%)	142 (8.7%)
Aetiology (PSC)	808 (10.5%)	145 (2.4%)	87 (5.3%)
Ascites	3285 (42.8%)	2972 (49%)	616 (37.6%)
No	1986 (25.8%)	970 (16%)	440 (26.9%)
Mild	2411 (31.4%)	2129 (35%)	582 (35.5%)
Moderate–severe			
Urea (mg/dL)	30.63 (IQR 23.42–42.64)	34 (IQR 26–47)	30.04 (IQR 24–48)
Creatinine (mg/dL)	0.91 ± 0.40	0.93 ± 0.49	0.95 ± 0.44
RFH‐GFR (ml/min)	69.81 ± 25.04	68.69 ± 24.02	66.51 ± 24.99
RFH‐GFR without ascites (ml/min)	71.39 ± 25.06	70.36 ± 23.81	68.17 ± 24.91
International normalised ratio	1.45 ± 0.44	1.43 ± 0.46	1.63 ± 0.59
Bilirubin (mg/dL)	2.57 (IQR 1.40–5.10)	1.80 (IQR 1–3.60)	3.10 (IQR 1.58–6.79)
Sodium (mmol/L)	136.24 ± 4.65	137.59 ± 4.77	136.17 ± 5.04
Albumin (g/dL)*	3.19 ± 0.66	3.48 ± 0.69	3.23 ± 0.69
MELD	15.03 ± 5.71	13.76 ± 5.76	16.94 ± 6.94
MELD‐Na	17.25 ± 6.44	15.61 ± 6.52	18.90 ± 7.65
MELD 3.0*	17.15 ± 6.29	14.99 ± 6.64	18.80 ± 7.59
GEMA‐Na	17.65 ± 5.84	16.30 ± 6.09	19.39 ± 6.94
GEMA‐Na without ascites	17.48 ± 5.76	16.11 ± 5.97	19.20 ± 6.85
Length in waiting‐list (only transplanted)	74 (IQR 24–199)	73 (IQR 23–174)	135 (IQR 50–307)
Primary outcome	449 (5.8%)	286 (4.7%)	87 (5.3%)
Transplanted	5895 (76.7%)	5187 (85.5%)	1307 (79.8%)
Death in waiting‐list	529 (6.9%)	189 (3.1%)	112 (6.8%)
Excluded due to clinical deterioration	430 (5.6%)	505 (8.3%)	121 (7.4%)

Abbreviations: GEMA‐Na, gender‐equity model for liver allocation corrected by serum sodium; MELD, model for end‐stage liver disease; MELD‐Na, model for end‐stage liver disease corrected by serum sodium; MELD 3.0, model for end‐stage liver disease 3.0; RFH‐GFR, royal free glomerular filtration rate.

* Serum albumin and MELD 3.0 were not available in 549 patients (3.6% of the whole cohort).

### 3.2 Performance of the RFH‐GFR Without Ascites in Assessing GFR

The presence of moderate–severe ascites decreases the RFH‐GFR by 8% according to the formula. The RFH‐GFR model accurately assessed GFR even without the inclusion of ascites. Full details are shown in the supporting information, Table S1 and Figures S1 and S2.

### 3.3 Prognostic Significance of Ascites Among Liver Transplant Candidates

The presence and grade of ascites at inclusion on the waiting‐list was associated with an incremental prevalence of 90‐day mortality or delisting for sickness. Full details are shown in the supporting information, Tables S2–S7 and Figure S3.

### 3.4 Discrimination of Predictive Models

The discrimination analysis including Hc statistics, 95% CI and *p* values for relevant comparisons between GEMA‐Na with and without ascites, and compared with MELD‐Na and MELD 3.0, is shown in Table 2. In the overall study population, removing ascites from the GEMA‐Na score resulted in a minimal decrease in its discriminative capacity to predict mortality or delisting due to clinical deterioration within the first 90 days: Hc = 0.755 (95% CI 0.735–0.774) vs. Hc = 0.753 (95% CI 0.733–0.773); *p* = 0.007. However, GEMA‐Na without ascites performed significantly better than MELD 3.0 (Hc = 0.734 [95% CI 0.714–0.754]; *p* < 0.001) and MELD‐Na (Hc = 0.737 [95% CI 0.717–0.757]; *p* < 0.001). In women, GEMA‐Na with and without ascites had comparable discrimination (Hc = 0.784 [95% CI 0.751–0.816] vs. Hc = 0.783 [95% CI 0.751–0.816]; *p* = 0.61), which was higher than that observed with MELD 3.0 (Hc = 0.750 [95% CI 0.716–0.784]; *p* < 0.001), and with MELD‐Na (Hc = 0.749 [95% CI 0.714–0.784]; *p* < 0.001). Among patients with moderate–severe ascites at inclusion on the waiting‐list, GEMA‐Na without ascites showed better discrimination than MELD 3.0 and MELD‐Na in the UK cohort (Hc = 0.763 vs. Hc = 0.735 [*p* < 0.001] and Hc = 0.743 [*p* = 0.002], respectively), Australian cohort (Hc = 0.764 vs. Hc = 0.736 [*p* = 0.018] and Hc = 0.718 [*p* = 0.016], respectively), and in the Spanish cohort (Hc = 0.734 vs. Hc = 0.717 [*p* = 0.06], and Hc = 0.717 [*p* = 0.019], respectively). The superiority of GEMA‐Na with or without ascites was more pronounced in patients with primary biliary cholangitis and primary sclerosing cholangitis (Table S8). The Brier scores showed similar overall accuracy of GEMA‐Na with or without ascites, which were higher than obtained by MELD 3.0 and MELD‐Na (Table S9).

**TABLE 2:** Harrells’ c statistics and 95% confidence intervals (in brackets) for each model in the overall cohort, and in the pre‐specified subgroups of interest to predict the primary outcome of the study. *p* values for comparing discrimination are shown for GEMA‐Na without ascites vs. GEMA‐Na (*), MELD 3.0 (**) and MELD‐Na (***).

COHORT	*n*	MELD‐Na	MELD 3.0	GEMA‐Na	GEMA‐Na without ascites	*p*
Overall cohort	14 842	0.737 (0.717–0.757)	0.734 (0.714–0.754)	0.755 (0.735–0.774)	0.753 (0.733–0.773)	*p = 0.007 **p < 0.001 ***p < 0.001
Overall cohort (women)	4160	0.749 (0.714–0.784)	0.750 (0.716–0.784)	0.784 (0.751–0.816)	0.783 (0.751–0.816)	*p = 0.61 **p < 0.001 ***p < 0.001
UK cohort	7133	0.766 (0.739–0.792)	0.758 (0.731–0.785)	0.786 (0.760–0.811)	0.784 (0.758–0.810)	*p = 0.09 **p < 0.001 ***p < 0.001
UK cohort (women)	2354	0.770 (0.725–0.815)	0.766 (0.721–0.810)	0.809 (0.769–0.849)	0.808 (0.768–0.848)	*p = 0.54 **p < 0.001 ***p < 0.001
UK cohort (moderate–severe ascites)	2256	0.743 (0.705–0.780)	0.735 (0.696–0.774)	0.763 (0.727–0.800)	0.763 (0.727–0.799)	*p = 0.88 **p < 0.001 ***p = 0.002
Australian cohort	1638	0.745 (0.690–0.800)	0.749 (0.696–0.802)	0.774 (0.720–0.827)	0.769 (0.716–0.823)	*p = 0.015 **p = 0.019 ***p = 0.028
Australian cohort (women)	432	0.714 (0.592–0.835)	0.732 (0.625–0.839)	0.796 (0.698–0.895)	0.792 (0.693–0.891)	*p = 0.21 **p = 0.008 ***p = 0.008
Australian cohort (moderate–severe ascites)	582	0.718 (0.641–0.795)	0.736 (0.665–0.807)	0.771 (0.704–0.838)	0.764 (0.696–0.832)	*p = 0.026 **p = 0.018 ***p = 0.016
Spanish cohort	6071	0.704 (0.670–0.738)	0.706 (0.672–0.740)	0.716 (0.682–0.750)	0.715 (0.680–0.749)	*p = 0.15 **p = 0.13 ***p = 0.06
Spanish cohort (women)	1374	0.742 (0.680–0.804)	0.745 (0.685–0.804)	0.755 (0.694–0.816)	0.757 (0.696–0.817)	*p = 0.40 **p = 0.36 ***p = 0.19
Spanish cohort (moderate–severe ascites)	2129	0.717 (0.668–0.766)	0.717 (0.668–0.766)	0.736 (0.690–0.782)	0.734 (0.687–0.780)	*p = 0.24 **p = 0.06 ***p = 0.019

*Note:* Patients with incomplete data to calculate MELD 3.0 were not included in the analysis (*n* = 549; 3.6%).

Abbreviations: GEMA‐Na, gender‐equity model for liver allocation corrected by serum sodium; MELD 3.0, model for end‐stage liver disease3.0; MELD‐Na, model for end‐stage liver disease corrected by serum sodium.

### 3.5 Calibration of GEMA‐Na Without Ascites and MELD 3.0

GEMA‐Na without ascites and MELD 3.0 had a comparable calibration profile across the different study cohorts. In patients with moderate–severe ascites, calibration was optimal in the UK and Spain: GEMA‐Na without ascites χ^2^ = 5.711 (*p* = 0.77) and χ^2^ = 8.753 (*p* = 0.46), respectively, and MELD 3.0 χ^2^ = 7.078 (*p* = 0.63) and χ^2^ = 7.672 (*p* = 0.56), respectively. Calibration was suboptimal in patients without moderate–severe ascites for both models in the UK and Spain: GEMA‐Na without ascites χ^2^ = 24.971 (*p* = 0.002) and χ^2^ = 15.953 (*p* = 0.043), respectively, and MELD 3.0 χ^2^ = 24.739 (*p* = 0.003) and χ^2^ = 17.341 (*p* = 0.044), respectively. The Australian cohort could not be stratified according to the presence of moderate–severe ascites due to insufficient number of patients but showed adequate calibration for both models: GEMA‐Na without ascites χ^2^ = 3.606 (*p* = 0.94), and MELD 3.0 χ^2^ = 6.720 (*p* = 0.35). Bar calibration plots are shown in Figures S4 and S5.

### 3.6 Differential Prioritisation

**FIGURE 1:**
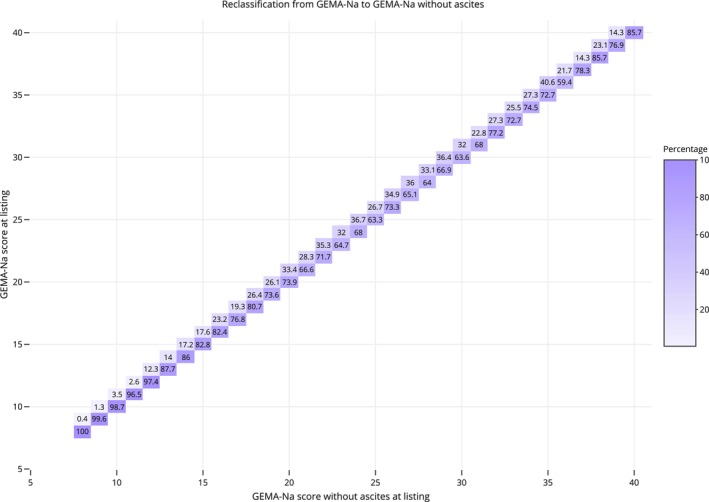
Re‐classification diagram showing patients’ prioritisation of GEMA‐Na with and without ascites in the whole study population. The number in each box represents the percentage of GEMA‐Na without ascites for a specific GEMA‐Na. The diagonal represents matching score values of both models. Above diagonal values represent the proportion of patients decreasing the score after removing ascites from a certain GEMA‐Na. There are no values below diagonal, meaning that no patient increased GEMA‐Na score after removing ascites. (two column fitting image).

In the whole study population, 2776 patients (18%) had a one‐point reduction in their allocation score after removing ascites from the GEMA‐Na equation, with an even distribution between men and women (18.2% vs. 17.6%; *p* = 0.34). The prevalence of a one‐point reduction in the GEMA‐Na score after removing ascites was 16.9% in the UK cohort, 18.9% in the Australian cohort, and 19.2% in the Spanish cohort (*p* = 0.002). Among 5121 patients with moderate–severe ascites, 54.2% of patients lost one point and 45.8% of patients retained the same score. No patient showed a difference of two or more score points. The distribution of patients showing a modification of the score after removing ascites across the GEMA‐Na range is shown in Figure 1. The likelihood of mortality or delisting for sickness within the first 90 days was higher in the group of patients who lost one point after removing ascites from GEMA‐Na compared with those who retained the same score (7.7% vs. 4.8%; *p* < 0.001). Conversely, the probability of transplantation was lower among patients who lost one point after removing ascites from the GEMA‐Na formula compared with those retaining the same score (74.7% vs. 77.8%; *p* = 0.006).

**FIGURE 2:**
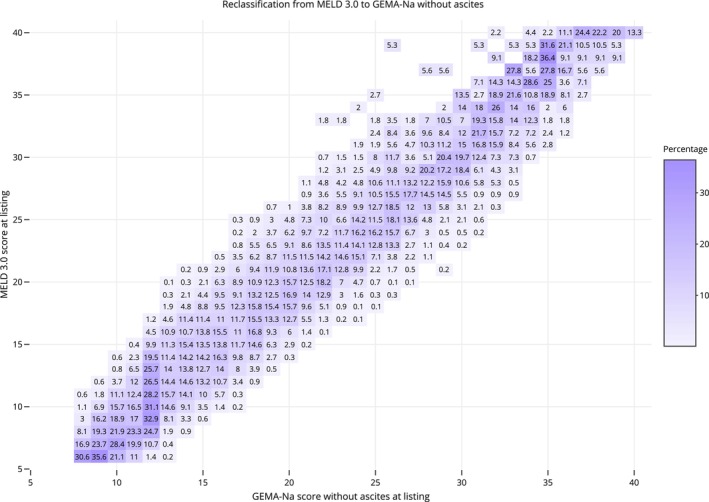
Re‐classification diagram showing patients’ prioritisation of GEMA‐Na without ascites and MELD 3.0 in the whole study population. The number in each box represents the percentage of GEMA‐Na without ascites for a specific MELD 3.0. The diagonal represents matching score values of both models. Above diagonal values represent the proportion of patients with higher MELD 3.0 score than GEMA‐Na without ascites score. Below diagonal values represent the proportion of patients with lower MELD 3.0 score than GEMA‐Na without ascites score. (two column fitting image).

**FIGURE 3:**
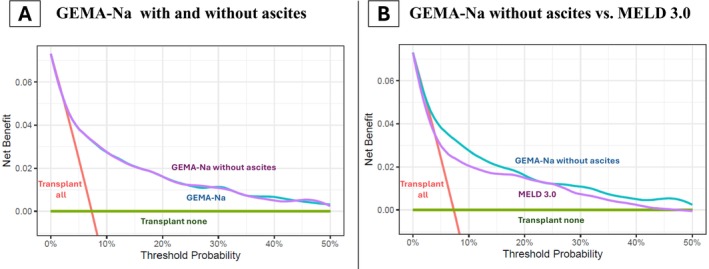
Decision curve analysis. Graphical plots of net benefit against the threshold probability of the primary outcome according to different strategies: Transplant all, transplant none, or transplant prioritisation according to evaluated models. Panel A: GEMA‐Na (blue) and GEMA‐Na without ascites (purple). Curves widely overlap indicating a comparable net benefit; Panel B: GEMA‐Na without ascites (blue) showed increased net benefit compared to MELD 3.0 (purple). (two column fitting image).

When transitioning from MELD 3.0 to GEMA‐Na without ascites, 12 853 patients (86.6%) changed the score by ≥ 1 point (54.4% upgraded, 29.1% downgraded), 8941 patients (60.3%) changed the score by ≥ 2 points (41.2% upgraded, 19.1% downgraded), and 5378 patients (36.3%) changed the score by ≥ 3 points (25% upgraded, 11.3% downgraded). The distribution of patients showing a modification of the score after transitioning from MELD 3.0 to GEMA‐Na without ascites is shown in Figure 2. According to the decision curve analysis, the clinical benefit was identical with GEMA‐Na with or without ascites, but it was superior with GEMA‐Na without ascites compared with MELD 3.0 (Figure 3).

## 4 Discussion

In the present study, which analysed data from over 15 000 candidates for elective LT across three countries with varying allocation systems and donation rates, we evaluated an iteration of GEMA‐Na that excludes ascites. We compared its performance against established liver allocation models, including MELD 3.0 and MELD‐Na. Our findings demonstrate that GEMA‐Na without ascites provides significantly better discriminatory ability than the MELD family scores, suggesting it may serve as a more effective tool for organ allocation in healthcare systems where incorporating ascites in prioritisation scores raises concerns about subjectivity.

Ascites is the most frequent decompensating event in cirrhosis. The presence of ascites is associated with frailty, impaired quality of life, and reduced energy and protein intake [20]. It is also associated with impaired renal function, due to the chronically reduced renal blood flow. In a retrospective study of 39 025 candidates for LT in the Organ Procurement and Transplantation Network database from 2016 to 2021, the presence of moderate–severe ascites was associated with two‐fold increase in waiting‐list mortality rates independently of the MELD 3.0 score, with a more pronounced increase among patients with MELD 3.0 lower than 30 [21]. Our results align with these findings, but we also observed an incremental risk of waiting‐list mortality according to the grade of ascites, and a more pronounced impact among women, which has not been previously described. The prognostic effect of moderate–severe ascites remained significant when controlling for MELD, MELD‐Na, or MELD 3.0, with an associated excess of risk of mortality or delisting for sickness ranging from 17% to 41%. However, the prognostic impact of moderate–severe ascites lost statistical significance when GEMA‐Na was included. Surprisingly, we observed a similar effect when considering GEMA‐Na without ascites. We hypothesized that the model may capture the true severity of patients with moderate‐to‐severe ascites, even when this information is not directly provided.

The small impact of ascites in the GEMA‐Na equation is not unexpected. Although moderate–severe ascites is included as a component in the RFH‐GFR formula, its weight is modest, as its presence reduces RFH‐GFR by only 8%. This reduction translates into an approximate increase of 0.692 points in the GEMA‐Na score, due to the logarithmic transformation of the RFH‐GFR within the equation. This theoretical effect was confirmed in the present study and had a negligible impact in the model's performance, including subgroups of interest such as patients with moderate–severe ascites and women. Therefore, GEMA‐Na with or without ascites could be used interchangeably in clinical practice but should not coexist within the same allocation system. Each transplant centre may decide which version of GEMA‐Na is to be used for all registrants. If several transplant centres share part of their waiting‐list for the sickest patients, all contributing centres may use the same version of GEMA‐Na to avoid heterogeneity in allocation.

The 2024 update of the EASL clinical practice guidelines on LT highlighted the significance of sex disparities in access to LT and the need to implement revised allocation systems to address these inequities [9]. Among these, MELD 3.0 and GEMA‐Na are the most robust. MELD 3.0 was developed and validated in the United States [10] while GEMA‐Na was trained in the UK and externally validated in Australia [11]. Although GEMA‐Na outperformed MELD 3.0 in several direct comparisons and has the potential to save lives among candidates for elective LT [6, 11, 12] its implementation is hindered by the need to record moderate–severe ascites. This study demonstrates, for the first time, that GEMA‐Na can be used without accounting for ascites and still outperforms MELD 3.0 and MELD‐Na. Among women, the discrimination of GEMA‐Na with and without ascites was identical and superior to MELD 3.0, suggesting that GEMA‐Na, even without ascites, would be equally effective in addressing sex disparities for LT access. It is reasonable to assume that the benefits of implementing GEMA‐Na, with or without ascites, would be more pronounced in countries with reduced donor availability.

The present study is limited by its retrospective nature. Although most variables, including waiting‐list outcomes, are prospectively recorded by official organ‐sharing organisations and subjected to periodic audits, data collection is vulnerable to heterogeneous practices as with other registries. The requirements of sample size were largely met in the UK and Spanish cohorts, but not in the Australian cohort in which non‐significant trends should be interpreted with caution. We used data from three different countries, allocation systems, and varying inclusion periods. However, we performed sensitivity analyses in each cohort with consistent results, which reinforced the external validity of the findings.

In conclusion, the presence and grade of ascites increase the risk of mortality or delisting due to clinical deterioration among candidates for elective LT. However, removing ascites from the GEMA‐Na calculation has only a marginal impact on the score and does not significantly affect the accuracy of its predictions. GEMA‐Na without ascites demonstrated superior discrimination compared with MELD 3.0 and MELD‐Na, including key subgroups of interest such as women and patients with moderate–severe ascites. These findings were consistent in three countries with diverse allocation systems and donation rates, supporting their broader applicability to other transplant settings.

## Author Contributions

**Manuel Luis Rodríguez‐Perálvarez:** study conception and design, obtained funding, data analysis and interpretation, drafting the manuscript and study guarantor. **Antonio Manuel Gómez‐Orellana, David Guijo‐Rubio, and César Hervás Martínez:** mathematical modelling, data analysis and interpretation, and critical revision of the article. **Avik Majumdar, Geoffrey W. McCaughan, María Victoria Aguilera, Mikel Gastaca, Carmen Cepeda‐Franco, María Luisa Ortiz, Jordi Colmenero, Alejandra Otero, Rocío González Grande, Alba Cachero, Esther Molina Pérez, Mónica Barreales, Rosa Martín**‐**Mateos, María Rodríguez‐Soler, Cristina Dopazo, Carmen Alonso Martín, Elena Otón, Luisa González Diéguez, María Dolores Espinosa, Ana Arias Milla, Gerardo Blanco Fernández, Sara Lorente, Antonio Cuadrado Lavín**, and **Miguel Sogbe:** data acquisition and critical revision of the article. **María Kalafateli:** data analysis and interpretation. **Rhiannon Taylor** and **Gloria de la Rosa:** data curation, analysis and critical revision of the article. **Emmanuel Tsochatzis:** study conception and design, manuscript writing, critical revision of the manuscript.

**Manuel Luis Rodríguez‐Perálvarez** and **Antonio Manuel Gómez‐Orellana:** had full access to the study data and verified the results. All authors revised and approved the final version of the article and had final responsibility for the decision to submit for publication.

## Funding

The present study was supported by the Spanish Ministry of Research and Innovation, the ‘Instituto de Salud Carlos III’ (grant reference PI22/00312), the Spanish Ministry of Science, Innovation and University (grant reference: PID2023‐150663NB‐C22), and co‐funded by the European Union. AMG‐O was supported by ‘Consejería de Transformación Económica, Industria, Conocimiento y Universidades de la Junta de Andalucía’ (grant reference: PREDOC‐00489). JC was supported by the Department de Recerca i Universitats de la Generalitat de Catalunya (codi expedient 2021‐SGR‐01331).

## Ethics Statement

Before sharing their corresponding anonymized databases, the study protocol was evaluated and approved by the NHS Blood and Transplant authority (UK), the ONT (Spain), and the Sydney Local Health District Human Research Ethics Committee (Australia). In addition, the study protocol was approved by the Andalusian ethics committee, which serves as the reference committee for the institution where the data analysis was conducted (reference 5408, 2022).

## Conflicts of Interest

M.L.R.‐P. has received lecture fees from Chiesi and Astellas Pharma, outside the present work. R.M.‐M. has received lecture fees from Chiesi, outside the present work. All other authors declare no competing interests.

## Supporting information


**Data S1:** Supporting Information.

## Data Availability

The data that support the findings of this study are available on request from the corresponding author. The data are not publicly available due to privacy or ethical restrictions.
