# Supplementary material for: Excluding Ascites From the GEMA‐Na Score Does Not Impact Outcome Predictions in Liver Transplant Candidates
Source: Liver Int. 2026 Jan 29;46(3):e70520. doi: 10.1111/liv.70520 (PMC12853322; doi:10.1111/liv.70520)
Supplement: Supplementary file 1 — Data S1: Supporting Information. [file LIV-46-0-s001.docx]

**SUPPLEMENTARY MATERIAL**

**Excluding ascites from the GEMA-Na score does not impact outcome predictions in liver transplant candidates.**

Manuel Luis Rodríguez Perálvarez, Antonio Manuel Gómez-Orellana, Avik Majumdar, Geoffrey W McCaughan, María Kalafateli, Rhiannon Taylor, Gloria de la Rosa, María Victoria Aguilera, Mikel Gastaca, Carmen Cepeda-Franco, José Antonio Pons, Jordi Colmenero, Alejandra Otero, Rocío González Grande, Alba Cachero, Esther Molina Pérez, Mónica Barreales, Rosa Martín Mateos, María Rodríguez-Soler, Mario Romero, Cristina Dopazo, Carmen Alonso Martín, Elena Otón, Luisa González Diéguez, María Dolores Espinosa, Ana Arias Milla, Gerardo Blanco Fernández, Sara Lorente, Antonio Cuadrado Lavín, Miguel Sogbe, David Guijo-Rubio, César Hervás Martínez, and Emmanuel Tsochatzis.

**TABLE OF CONTENTS**

- Supplementary material to sections 2.5 and 3.2, supplementary table 1 and supplementary figures 1-2 --------------------------------------------------------------- p. 3-5

- Supplementary material to section 3.3, supplementary tables 2-7, and supplementary figure 3 -------------------------------------------------------------------------------------- p. 6-9

- Supplementary table 8 ------------------------------------------------------------------- p. 10

- Supplementary table 9 ------------------------------------------------------------------- p. 11

- Supplementary figure 4 ------------------------------------------------------------------ p. 12

- Supplementary figure 5 ------------------------------------------------------------------ p. 13

- References --------------------------------------------------------------------------------- p. 14

**Performance of the Royal Free Hospital Glomerular Filtration Rate (RFH-GFR) without ascites to assess renal function in patients with cirrhosis.**

We tested an iteration of the RFH-GFR without ascites in the same population where the original formula was trained and internally validated.^1^ A consecutive cohort of patients with cirrhosis evaluated for liver transplantation at the Royal Free Hospital were included: 469 patients in the training cohort (2011-2014), and 174 patients in the internal validation cohort (2007-2010). The RFH-GFR without ascites was compared with a direct measurement of GFR using radioisotope plasma clearance. We calculated the coefficient of determination (r^2^ statistic), the mean difference between observed and predicted GFR (residual) values, the root mean square error (standard deviation of the mean difference), and the appropriate residual plots. Precision was assessed as interquartile range (IQR) for the difference. Accuracy was assessed as the percentage of predictions within 10% (P10), 30% (P30), and 50% (P50) of measured GFR. Confidence intervals of median difference, IQR, and P10, P30, and P50 were estimated with the bootstrap method (200 bootstraps). Significance testing was two-sided and set to <0.05. Analysis was performed using the SPSS statistical package (version 29.0.2.0).

The median RFH-GFR without ascites in the training and internal validation cohorts were 68.49 ml/min (95% CI: 66.55-70.73) and 73.04 ml/min (95% CI: 68.68-75.74), respectively. The mean difference between observed and predicted GFR (residual) values in the internal validation group was 2.52 mL/minute/1.73 m2 with a root mean square error of 15.018. Further details are shown in table I.

|  | **RFH-GFR (without ascites)** |
| --- | --- |
|  | **Training cohort** |
| Median difference (95% CI), ml/minute/1.73 m^2^ | -2.82 (-4.29, -0.79) |
| Interquartile Range for differences (95% CI), ml/minute/1.73 m^2^ | 17.85 (16.23, 19.28) |
| P10 (%), (95% CI) | 42.2 (38.0-47.3) |
| P30 (%), (95% CI) | 84.9 (81.4-88.2) |
| P50 (%), (95% CI) | 94.7 (92.5-96.9) |
|  | **Internal validation cohort** |
| Median difference (95% CI), ml/minute/1.73 m^2^ | 2.89 (-0.62, 3.90) |
| Interquartile Range for differences (95% CI), ml/minute/1.73 m^2^ | 21.71 (17.70, 26.10) |
| P10 (%), (95% CI) | 45.8 (38.4-54.2) |
| P30 (%), (95% CI) | 86.3 (79.8 -90.6) |
| P50 (%), (95% CI) | 93.7 (90.6-96.9) |

**Supplementary table 1.**

According to the residual plot (a) and Bland-Altman plot (b), the RFH-GFR without ascites had a good fit (supplementary figure 1).

**
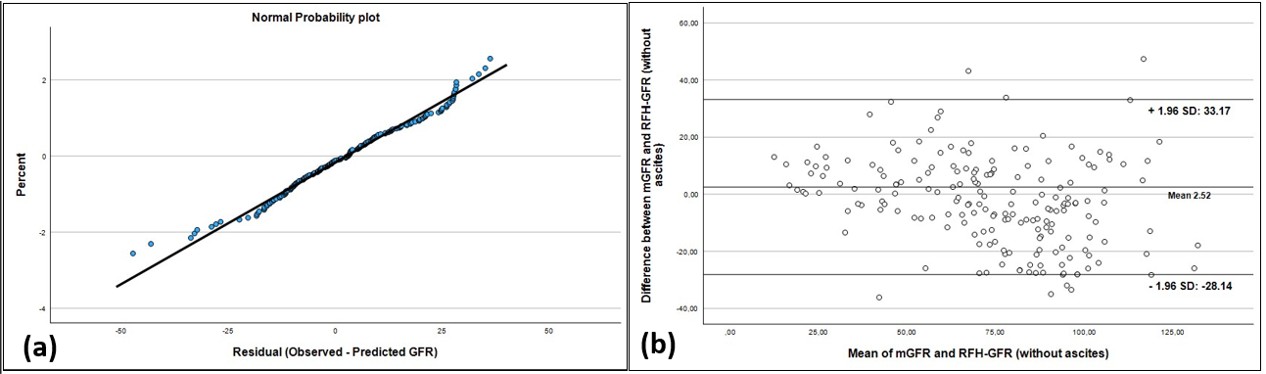
Supplementary figure 1.**

In the scatter/dot plot of estimated GFR for RFH-GFR (without ascites) compared with measured GFR using radioisotope plasma clearance, the residuals appear to be randomly scattered around zero, confirming a good fit.

**
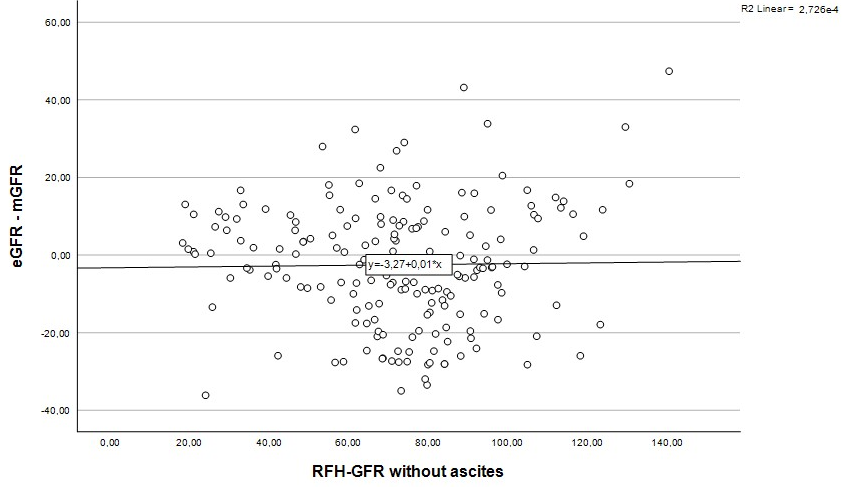
Supplementary figure 2.**

Therefore, we concluded that RFH-GFR without ascites still provides an accurate estimation of renal function.

**Prognostic significance of ascites among liver transplant candidates.**

The presence and grade of ascites at inclusion in the waiting list was associated with an incremental prevalence of 90-days mortality or delisting for sickness in the whole study population: 3.3% among patients without ascites, 5.8% in patients with mild ascites, and 7.7% in patients with moderate-severe ascites (p<0.001). Kaplan-Meier curves showing the cumulative incidence of the primary outcome according to the presence and grade of ascites in the whole study cohort are shown in the supplementary figure 3.

**Supplementary figure 3.**


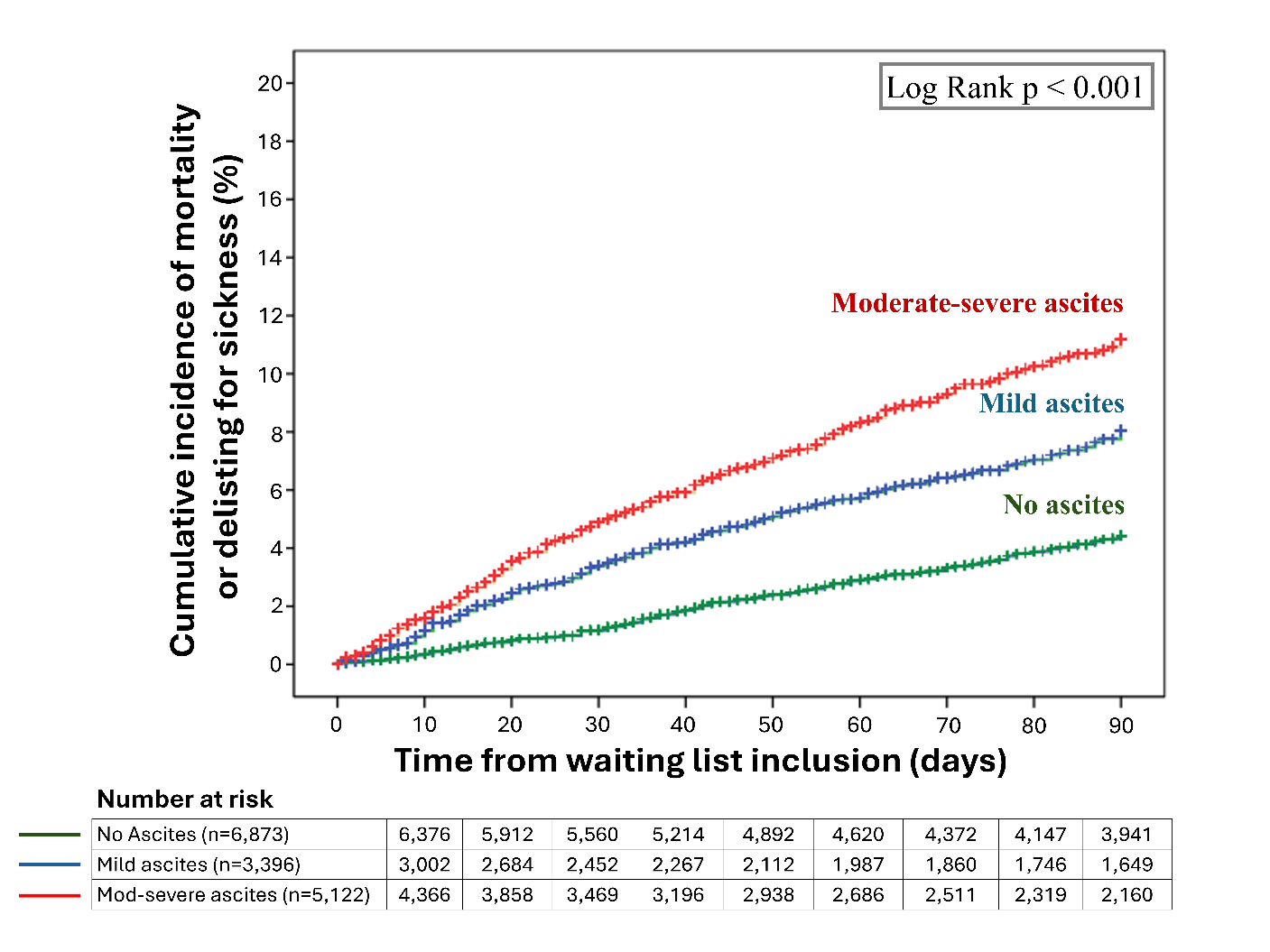


In the multivariable analysis after controlling for age, sex, aetiology of liver disease, and analytical parameters, the presence of moderate-severe ascites was an independent predictor of mortality or delisting due to clinical deterioration within the first 90 days (HR=1.42, 95%CI 1.22-1.66; p<0.001) as shown in supplementary table 2.

**Supplementary table 2.** Multivariable Cox’s regression analysis of clinical and analytical predictors determined at inclusion in the waiting list to predict mortality or delisting for sickness within the first 90 days in the whole study population (n=14,842)*.

| **Variable** | **β coefficient** | **HR** | **95%CI** | **p** |
| --- | --- | --- | --- | --- |
| Age | 0.025 | 1.025 | 1.017-1.033 | <0.001 |
| Sex (women) | 0.146 | 1.157 | 0.983-1.362 | 0.08 |
| Aetiology (hepatitis C) | -0.104 | 0.901 | 0.735-1.104 | 0.31 |
| Aetiology (alcohol) | 0.004 | 1.004 | 0.841-1.198 | 0.96 |
| Aetiology (MASH/cryptogenic) | 0.098 | 1.103 | 0.878-1.385 | 0.40 |
| Aetiology (PBC) | -0.298 | 0.742 | 0.531-1.038 | 0.74 |
| Aetiology (PSC) | -0.285 | 0.752 | 0.525-1.078 | 0.12 |
| Ascites (moderate/severe) | 0.353 | 1.423 | 1.222-1.657 | <0.001 |
| International normalized ratio | 0.470 | 1.600 | 1.431-1.789 | <0.001 |
| Serum bilirubin (mg/dL) | 0.071 | 1.074 | 1.066-1.081 | <0.001 |
| Serum sodium (mmol/L) | -0.058 | 0.944 | 0.931-0.956 | <0.001 |
| Serum albumin (g/dL) | -0.108 | 0.898 | 0.803-1.004 | 0.058 |
| Serum creatinine (mg/dL) | 0.224 | 1.251 | 1.126-1.391 | <0.001 |

*549 patients were excluded from this analysis due to missing serum albumin.

MASH: metabolic-associated steatohepatitis; PBC: primary biliary cholangitis; PSC: primary sclerosing cholangitis.

The impact of ascites on mortality or delisting for sickness at 90 days was significant both in men (HR=1.28, 95%CI 1.06-1.54; p=0.009) and in women (HR=1.86, 95%CI 1.42-2.44; p<0.001) in the multivariable analysis.

Of note, when prioritization scores were sequentially included in the multivariable analysis instead of their analytical components, moderate-severe ascites was an independent predictor of the primary outcome in multivariable models containing MELD (HR=1.41, 95%CI 1.22-1.63; p<0.001), MELD-Na (HR=1.16, 95%CI 1.01-1.35; p=0.038), and MELD 3.0 (HR=1.27, 95%CI 1.09-1.47; p=0.002). However, the impact of ascites lost statistical significance when GEMA-Na, either with (HR=0.99, 95%CI 0.85-1.14; p=0.84) or without ascites (HR=1.07, 95%CI 0.93-1.24; p=0.34) was analysed, as shown in supplementary tables 3-7.

**Supplementary table 3.** Multivariable Cox’s regression analysis of clinical and analytical predictors determined at inclusion in the waiting list to predict mortality or delisting for sickness within the first 90 days in the whole study population (n=15,391) when considering the model for end-stage liver disease (MELD).

| **Variable** | **β coefficient** | **HR** | **95%CI** | **p** |
| --- | --- | --- | --- | --- |
| Age | 0.024 | 1.024 | 1.016-1.031 | <0.001 |
| Sex (women) | 0.120 | 1.127 | 0.964-1.317 | 0.13 |
| Aetiology (hepatitis C) | -0.108 | 0.898 | 0.738-1.093 | 0.28 |
| Aetiology (alcohol) | -0.204 | 0.815 | 0.690-0.964 | 0.017 |
| Aetiology (MASH/cryptogenic) | -0.029 | 0.971 | 0.781-1.207 | 0.79 |
| Aetiology (PBC) | -0.314 | 0.730 | 0.531-1.005 | 0.05 |
| Aetiology (PSC) | -0.500 | 0.606 | 0.427-0.862 | 0.005 |
| Ascites (moderate/severe) | 0.342 | 1.408 | 1.220-1.625 | <0.001 |
| MELD | 0.144 | 1.155 | 1.144-1.167 | <0.001 |

MASH: metabolic-associated steatohepatitis; PBC: primary biliary cholangitis; PSC: primary sclerosing cholangitis; MELD: model for end-stage liver disease.

**Supplementary table 4.** Multivariable Cox’s regression analysis of clinical and analytical predictors determined at inclusion in the waiting list to predict mortality or delisting for sickness within the first 90 days in the whole study population (n=15,391) when considering the model for end-stage liver disease corrected by serum sodium (MELD-Na).

| **Variable** | **β coefficient** | **HR** | **95%CI** | **p** |
| --- | --- | --- | --- | --- |
| Age | 0.022 | 1.022 | 1.015-1.030 | <0.001 |
| Sex (women) | 0.111 | 1.117 | 0.956-1.306 | 0.16 |
| Aetiology (hepatitis C) | -0.127 | 0.881 | 0.724-1.072 | 0.20 |
| Aetiology (alcohol) | -0.252 | 0.777 | 0.657-0.919 | 0.003 |
| Aetiology (MASH/cryptogenic) | -0.056 | 0.945 | 0.760-1.176 | 0.61 |
| Aetiology (PBC) | -0.340 | 0.712 | 0.517-0.980 | 0.037 |
| Aetiology (PSC) | -0.468 | 0.626 | 0.441-0.890 | 0.009 |
| Ascites (moderate/severe) | 0.153 | 1.166 | 1.008-1.348 | 0.038 |
| MELD-Na | 0.148 | 1.159 | 1.147-1.172 | <0.001 |

MASH: metabolic-associated steatohepatitis; PBC: primary biliary cholangitis; PSC: primary sclerosing cholangitis; MELD-Na: model for end-stage liver disease corrected by serum sodium.

**Supplementary table 5.** Multivariable Cox’s regression analysis of clinical and analytical predictors determined at inclusion in the waiting list to predict mortality or delisting for sickness within the first 90 days in the whole study population (n=14,842)* when considering the model for end-stage liver disease 3.0 (MELD 3.0).

| **Variable** | **β coefficient** | **HR** | **95%CI** | **p** |
| --- | --- | --- | --- | --- |
| Age | 0.027 | 1.028 | 1.020-1.036 | <0.001 |
| Sex (women) | -0.073 | 0.929 | 0.790-1.093 | 0.38 |
| Aetiology (hepatitis C) | -0.066 | 0.936 | 0.764-1.147 | 0.53 |
| Aetiology (alcohol) | -0.125 | 0.883 | 0.742-1.051 | 0.16 |
| Aetiology (MASH/cryptogenic) | 0.002 | 1.002 | 0.800-1.256 | 0.99 |
| Aetiology (PBC) | -0.244 | 0.783 | 0.564-1.089 | 0.15 |
| Aetiology (PSC) | -0.359 | 0.698 | 0.489-0.997 | 0.048 |
| Ascites (moderate/severe) | 0.237 | 1.268 | 1.092-1.472 | 0.002 |
| MELD 3.0 | 0.143 | 1.154 | 1.142-1.166 | <0.001 |

*549 patients were excluded from this analysis due to missing serum albumin

MASH: metabolic-associated steatohepatitis; PBC: primary biliary cholangitis; PSC: primary sclerosing cholangitis; MELD 3.0: model for end-stage liver disease 3.0.

**Supplementary table 6.** Multivariable Cox’s regression analysis of clinical and analytical predictors determined at inclusion in the waiting list to predict mortality or delisting for sickness within the first 90 days in the whole study population (n=15,391) when considering the gender-equity model for liver allocation corrected by serum sodium (GEMA-Na).

| **Variable** | **β coefficient** | **HR** | **95%CI** | **p** |
| --- | --- | --- | --- | --- |
| Age | 0.014 | 1.014 | 1.007-1.022 | <0.001 |
| Sex (women) | -0.031 | 0.969 | 0.829-1.132 | 0.69 |
| Aetiology (hepatitis C) | -0.127 | 0.881 | 0.724-1.071 | 0.20 |
| Aetiology (alcohol) | -0.239 | 0.787 | 0.666-0.931 | 0.005 |
| Aetiology (MASH/cryptogenic) | -0.069 | 0.934 | 0.751-1.161 | 0.54 |
| Aetiology (PBC) | -0.307 | 0.735 | 0.534-1.012 | 0.06 |
| Aetiology (PSC) | -0.381 | 0.684 | 0.481-0.971 | 0.034 |
| Ascites (moderate/severe) | 0.015 | 0.985 | 0.850-1.141 | 0.84 |
| GEMA-Na | 0.167 | 1.182 | 1.168-1.195 | <0.001 |

MASH: metabolic-associated steatohepatitis; PBC: primary biliary cholangitis; PSC: primary sclerosing cholangitis; GEMA-Na: gender-equity model for liver allocation corrected by serum sodium.

**Supplementary table 7.** Multivariable Cox’s regression analysis of clinical and analytical predictors determined at inclusion in the waiting list to predict mortality or delisting for sickness within the first 90 days in the whole study population (n=15,391) when considering the gender-equity model for liver allocation corrected by serum sodium (GEMA-Na) without ascites.

| **Variable** | **β coefficient** | **HR** | **95%CI** | **p** |
| --- | --- | --- | --- | --- |
| Age | 0.014 | 1.014 | 1.007-1.021 | <0.001 |
| Sex (women) | -0.028 | 0.973 | 0.833-1.137 | 0.73 |
| Aetiology (hepatitis C) | -0.126 | 0.881 | 0.725-1.072 | 0.21 |
| Aetiology (alcohol) | -0.235 | 0.791 | 0.669-0.935 | 0.006 |
| Aetiology (MASH/cryptogenic) | -0.065 | 0.937 | 0.753-1.165 | 0.56 |
| Aetiology (PBC) | -0.305 | 0.737 | 0.563-1.015 | 0.06 |
| Aetiology (PSC) | -0.381 | 0.683 | 0.480-0.971 | 0.033 |
| Ascites (moderate/severe) | 0.071 | 1.073 | 0.927-1.242 | 0.34 |
| GEMA-Na without ascites | 0.166 | 1.180 | 1.167-1.194 | <0.001 |

MASH: metabolic-associated steatohepatitis; PBC: primary biliary cholangitis; PSC: primary sclerosing cholangitis; GEMA-Na: gender-equity model for liver allocation corrected by serum sodium.

**Supplementary table 8.** Harrells’ c statistics and 95% confidence intervals (in brackets) for each model according to the most frequent aetiologies of liver disease motivating liver transplantation. Only patients with data available to calculate all models were included. P values for comparing discrimination are shown for GEMA-Na without ascites vs. GEMA-Na (*), MELD 3.0 (**) and MELD-Na (***).

| **AETIOLOGY** | **n** | **MELD-Na** | **MELD 3.0** | **GEMA-Na** | **GEMA-Na without ascites** | **p** |
| --- | --- | --- | --- | --- | --- | --- |
| Alcohol-related liver disease | 6,169 | 0.716  (0.685-0.747) | 0.714  (0.682-0.745) | 0.732  (0.701-0.763) | 0.728  (0.697-0.759) | *p=0.002  **p=0.006  ***p=0.011 |
| MASH/cryptogenic | 1,615 | 0.757  (0.703-0.810) | 0.754  (0.700-0.808) | 0.776  (0.725-0.827) | 0.773  (0.722-0.824) | *p=0.27  **p=0.10  ***p=0.13 |
| Hepatitis C | 3,167 | 0.720  (0.671-0.769) | 0.714  (0.665-0.762) | 0.723  (0.673-0.762) | 0.722  (0.672-0.773) | *p=0.57  **p=0.30  ***p=0.93 |
| Primary biliary cholangitis | 906 | 0.770  (0.691-0.848) | 0.763  (0.685-0.840) | 0.796  (0.723-0.870) | 0.796  (0.723-0.870) | *p=0.99  **p=0.028  ***p=0.029 |
| Primary sclerosing cholangitis | 999 | 0.774  (0.687-0.860) | 0.759  (0.671-0.848) | 0.811  (0.730-0.892) | 0.803  (0.721-0.885) | *p=0.036  **p=0.009  ***p=0.002 |

MASH: metabolic-associated steatohepatitis.

**Supplementary table 9.** Brier scores calculated as mean squared error of predicted probabilities of each prioritization score in the overall study population, and in each cohort and subgroups of interest separately. Lower Brier scores indicate better overall accuracy of the model.

| **COHORT** | **n** | **MELD-Na** | **MELD 3.0** | **GEMA-Na** | **GEMA-Na without ascites** |
| --- | --- | --- | --- | --- | --- |
| Overall cohort | 14,842 | 0.0521 | 0.0524 | 0.0506 | 0.0507 |
| Overall cohort (Women) | 4,160 | 0.0530 | 0.0535 | 0.0502 | 0.0504 |
| UK cohort | 7,133 | 0.0528 | 0.0533 | 0.0509 | 0.0508 |
| UK cohort (women) | 2,354 | 0.0521 | 0.0523 | 0.0482 | 0.0482 |
| UK cohort  (moderate-severe ascites) | 2,256 | 0.0749 | 0.0762 | 0.0735 | 0.0731 |
| Australian cohort | 1,638 | 0.0474 | 0.0472 | 0.0454 | 0.0457 |
| Australian cohort (women) | 432 | 0.0493 | 0.0491 | 0.0452 | 0.0456 |
| Australian cohort (moderate-severe ascites) | 582 | 0.0747 | 0.0738 | 0.0694 | 0.0699 |
| Spanish cohort | 6,071 | 0.0517 | 0.0517 | 0.0507 | 0.0508 |
| Spanish cohort (women) | 1,374 | 0.0540 | 0.0539 | 0.0529 | 0.0530 |
| Spanish cohort (moderate-severe ascites) | 2,129 | 0.0711 | 0.0715 | 0.0694 | 0.0698 |

Footnote: MELD-Na: Model for end-stage liver disease corrected by serum sodium; MELD 3.0: Model for end-stage liver disease3.0; GEMA-Na: Gender-Equity model for liver allocation corrected by serum sodium. Patients with incomplete data to calculate MELD 3.0 were not included in the analysis (n=549; 3.6%).


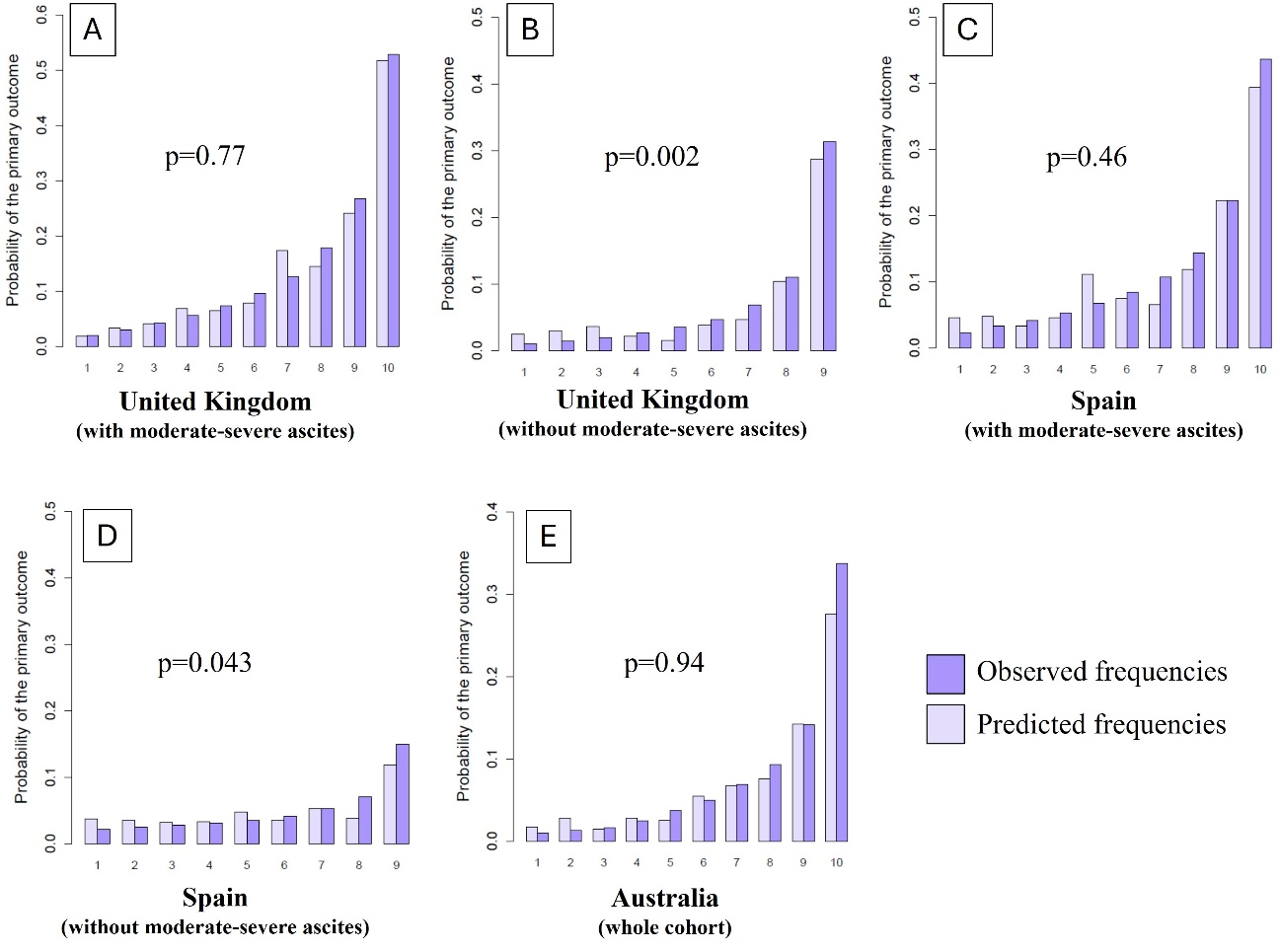


**Supplementary figure 4.** Bar-calibration plots of the Gender-Equity Models for liver Allocation corrected with serum sodium (GEMA-Na) without ascites across different study cohorts. The predicted and observed probabilities for the primary outcome are presented. Each cohort was stratified into deciles of risk, which were merged if needed to ensure a minimum of five events per group of risk (X axis). P values correspond to the Greenwood-Nam-D’Agostino goodness-of-fit test. (A) United Kingdom, patients with moderate-severe ascites (n = 2,410); (B) United Kingdom, patients without moderate-severe ascites (n = 5,272); (C) Spain, patients with moderate-severe ascites (n = 2,129); (D) Spain, patients without moderate-severe ascites (n = 3,942); (E) Australia, whole cohort (n = 1,638).

**
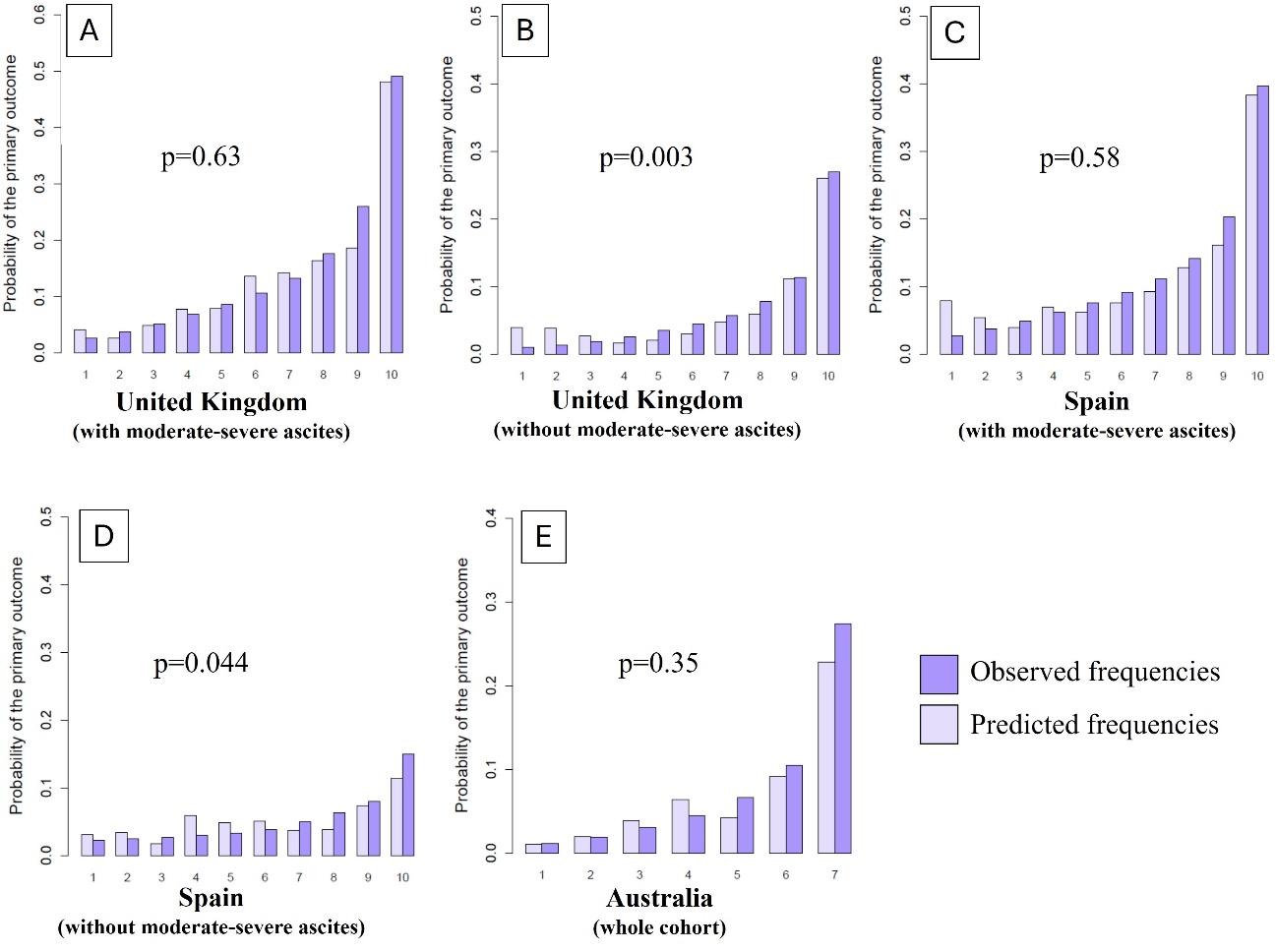
**

**Supplementary figure 5.** Bar-calibration plots of the Model for End-stage Liver Disease 3.0 (MELD 3.0) across different study cohorts. The predicted and observed probabilities for the primary outcome are presented. Each cohort was stratified into deciles of risk, which were merged if needed to ensure a minimum of five events per group of risk (X axis). P values correspond to the Greenwood-Nam-D’Agostino goodness-of-fit test. (A) United Kingdom, patients with moderate-severe ascites (n = 2,410); (B) United Kingdom, patients without moderate-severe ascites (n = 5,272); (C) Spain, patients with moderate-severe ascites (n = 2,129); (D) Spain, patients without moderate-severe ascites (n = 3,942); (E) Australia, whole cohort (n = 1,638).

**REFERENCES**

[1] Royston P, Sauerbrei W. A new measure of prognostic separation in survival data. Stat Med 2004;23:723-748.
